# Characterization of the Largest Secretory Protein Family, Ricin B Lectin-like Protein, in *Nosema bombycis*: Insights into Microsporidian Adaptation to Host

**DOI:** 10.3390/jof8060551

**Published:** 2022-05-24

**Authors:** Jinzhi Xu, Jian Luo, Jiajing Chen, Charles R. Vossbrinck, Tian Li, Zeyang Zhou

**Affiliations:** 1 State Key Laboratory of Silkworm Genome Biology, Southwest University, Chongqing 400715, China; xjz_swu@126.com (J.X.); jianluo0214@163.com (J.L.); chen9710262022@163.com (J.C.); 2 Chongqing Key Laboratory of Microsporidia Infection and Control, Southwest University, Chongqing 400715, China; 3 Department of Environmental Science, The Connecticut Agricultural Experiment Station, 123 Huntington Street, New Haven, CT 06511, USA; charles.vossbrinck@ct.gov; 4 College of Life Science, Chongqing Normal University, Chongqing 400047, China

**Keywords:** microsporidia, *Nosema bombycis*, Ricin B lectin-like, secreted protein, evolution

## Abstract

Microsporidia are a group of obligate intracellular pathogens infecting nearly all animal phyla. The microsporidian *Nosema bombycis* has been isolated from several lepidopteran species, including the economy-important silkworms as well as several crop pests. Proteins secreted by parasites can be important virulent factors in modulating host pathways. Ricin is a two-chain lectin best known for its extreme vertebrate toxicity. Ricin B lectin-like proteins are widely distributed in microsporidia, especially in *N. bombycis.* In this study, we identify 52 Ricin B lectin-like proteins (RBLs) in *N. bombycis*. We show that the *N. bombycis* RBLs (NbRBLs) are classified into four subfamilies. The subfamily 1 was the most conserved, with all members having a Ricin B lectin domain and most members containing a signal peptide. The other three subfamilies were less conserved, and even lost the Ricin B lectin domain, suggesting that NbRBLs might be a multi-functional family. Our study here indicated that the NbRBL family had evolved by producing tandem duplications firstly and then expanded by segmental duplications, resulting in concentrated localizations mainly in three genomic regions. Moreover, based on RNA-seq data, we found that several *Nbrbl*s were highly expressed during infection. Further, the results show that the NbRBL28 was secreted into host nucleus, where it promotes the expressions of genes involved in cell cycle progression. In summary, the great copy number, high divergence, and concentrated genome distribution of the NbRBLs demonstrated that these proteins might be adaptively evolved and played a vital role in the multi-host *N. bombycis*.

## 1. Introduction

Microsporidia are a group of obligate intracellular parasites, which can infect a wide variety of hosts from protists to mammals, even humans [1,2,3,4]. *Nosema bombycis*, a kind of parasite of the silkworm (*Bombyx mori*), was the first formally described microsporidia and has been shown to be transmitted both vertically and horizontally. Infection of *N. bombycis* will result in the death of its host, thus posing a significant threat to sericulture industry [5]. As intracellular parasites, microsporidia utilize many of its host metabolites to reduce genomes and thus speed up reproduction [6,7].

Ricin, notable for its extreme vertebrate toxicity, is a heterodimeric protein (carbohydrate containing protein) and is found in the seeds of the castor oil plant *Ricinus communis* L [8]. It has a cell-binding ricin toxic B chain (RTB) linked, through a disulfide bound, to a catalytic cytotoxic ribosome-inactivating protein (RTA). The RTB has two sugar-binding regions, each of which contain three homologous subregions (alpha, beta, and gamma) composed of 40 amino acids and a linker peptide around 15 residues (lambda). It has been proposed that RTB originated from a primitive 40 residue galactoside-binding peptide, which evolved through gene duplication and expansion [9,10]. Ricin B-lectin is homologous to the RTB and usually contains a conserved Ricin B-lectin domain. RTB can bind with the exposed galactose residues of multiple glycoproteins and glycolipids on cell surface [11]. Lectins such as RTB have a wide range of receptor-binding capabilities and are an important pathogenic factor in host–pathogens interactions. They mediate intercellular adhesion, infection, natural immune defense, and host-phagocytic action to eliminate invading pathogens, but the mechanisms remain unclear [12,13].

Previous studies showed that Ricin B lectin-like (RBL) in *Encephalitozoon cuniculi* and *Nosema ceranae* could form genomic clusters. In *E. cuniculi*, four RBL coding genes (*rbls*) were found in one cluster. In *N. ceranae*, six *rbls* sit together in a genomic region. In particular, no *rbl* was found in *Nematocida parisii*, suggesting that the family may have been lost during genome reduction. Experimental studies have demonstrated that RBL is produced during the germination of *Spraguea lophii* spores, indicating that these proteins might promote infection or resist host immunity [14]. Previous studies have shown that microsporidian RBLs (EcRBLL-1, AaRBLL-1, AaRBLL-2, and NbRBL) enhance spore adhesion to host cells [15,16]. All these findings suggest that this protein family is vital during microsporidian infection.

In this study, we identified the *N. bombycis* Ricin B lectin-like proteins (NbRBLs), and characterized their sequence and evolution, as well as gene expression patterns during infection.

## 2. Materials and Methods

### 2.1. Prediction and Identification of NbRBLs

Protein sequences of *N. bombycis* were downloaded from the SilkPathDB [17]. The hidden Markov model (HMM) profile of the RBL domain (Pfam: PF00652) was downloaded from the Pfam [18] and it was utilized to search the candidate RBLs from *N. bombycis* protein sequences. The candidate sequences were then used to searched against *N. bombycis* genome to detect missed RBLs using BLASTP [19] with an e-value less than 0.05. Two online programs, SMART (http://smart.embl-heidelberg.de/, accessed on 3 June 2019) and CDD (http://www.ncbi.nlm.nih.gov/Structure/cdd/wrpsb.cgi, accessed on 3 June 2019), were used to characterize the domain architecture of the predicted NbRBLs.

### 2.2. Sequence Features of NbRBLs

Sequence features of the candidate NbRBLs were predicted using the Protparam tool [20]. The subcellular localization of NbRBLs was predicted by two programs: WoLF PSORT [21] and TargetP (http://www.cbs.dtu.dk/services/TargetP, accessed on 3 August 2020). Signal peptides (SPs) were predicted by SignalP 5.0 [22] with the default D-cutoff values (https://services.healthtech.dtu.dk/service.php?SignalP-5.0, accessed on 5 August 2020).

Conserved motifs of the SP were identified using MEME 5.3.0 [23] with default parameters, except for the maximum number of motifs and maximum width, which were set to 4 and 10, respectively. Only motifs with an *e* value < 1e^−22^ were kept for further analysis.

### 2.3. Multiple Sequence Alignment and Phylogenetic Analysis of NbRBLs

Multiple protein sequences were aligned using Clustal Omega (https://www.ebi.ac.uk/Tools/msa/clustalo/, accessed on 3 August 2020), the results of which were then plotted using the online BoxShade (https://embnet.vital-it.ch/software/BOX_form.html, accessed on 3 August 2020). 

To investigate the evolutionary history of the NbRBLs, phylogenetic trees were constructed using the Maximum Likelihood (ML) method in RaxML [24] with a bootstrap test for 500 replicates and visualized by iTOL (http://itol.embl.de/, accessed on 15 September 2021) [25,26], using the castor RICOM_RicinB (GenBank accession no, ACY38598.1) as an outer group.

### 2.4. Expressions of Nbrbls during N. bombycis Infection

*N. bombycis* spores were prepared as previously described [27]. The spores, which were pretreated with 0.1 mol/L KOH, were added to the BmE-SWU1 cells (cell:spore ratio, 1:20). Infected cells were collected at 12-, 24-, 48-, 60- and 72-h post infection (hpi) and stored in TRIzol (Ambion, CA, USA). RNA extraction and cDNA synthesis were performed as previously described [28]. The real-time quantitative PCR (RT-qPCR) was conducted using primers (Nbrbl16, Nbrbl45, and Nbrbl51) and reference gene *Nbtubulin* (Table 1). Expression levels were calculated by the 2^−ΔΔt^ values method using three replicates. All statistical *t*-tests were performed with GraphPad Prism version 9.0.0 by two-tailed comparison tests and any difference with a *p*-value < 0.05 was considered significant [29].

Transcriptomic data were downloaded from scientific publications (the accession number PRJNA549766) as reference and were used to analyze the expression patterns of NbRBL proteins [30].

### 2.5. Indirect Immunofluorescence Assay (IFA)

Infected *BmE-SWU1* cells were fixed with 4% paraformaldehyde for 10 min at room temperature and washed three times with 1xPBS and permeabilized using 0.1% Triton X-100 for 15 min. The cells were then blocked in 1xPBST containing 5% BSA and 10% goat serum for 1 h at room temperature. Next, the cells were incubated with mouse and rabbit poly clonal antibodies against NbRBL06 (anti-NbRBL06) and NbRBL28 (anti-NbRBL28) diluted 1:100 in blocking solution for 2 h at room temperature. The cells were then washed for three times with 1xPBST, and incubated for 1 h with a 1:1000 dilution of Alexa Fluor 488 conjugate Goat anti-Mouse IgG (Invitrogen A32723, Rockford, Illinois, USA) and Alexa Fluor 594 conjugate Goat anti- Mouse IgG (Invitrogen A32742, Rockford, Illinois, USA) in a dark moist chamber at room temperature. The cell nucleus was stained with DAPI (1:1000 dilution, Sigma-Aldrich 28718-90-3, St. Louis, MO, USA) at room temperature for 15 min. The samples were finally observed and photographed using an Olympus FV1200 laser scanning confocal microscope.

### 2.6. Transfection and RNA-seq

The *Nbrbl28* (locus NBO_163g0001) was cloned from *N. bombycis* genomic DNA and inserted into the pSL1180 over expression vector fused with *egfp*. BmE-SWU1 cells were transfected with *Nbrbl28::egfp-* and *egfp-*containing plasmids according to the instructions of X-tremeGENE HP DNA transfection reagents (Roche 06366546001). The cell culture medium was replaced with fresh Grace’s Insect medium containing 10% serum after 5 h. Three days later, the transfected cells were collected, and stored at −80 °C for RNA-seq. RNA-seq was conducted by the Biomarker Technology Company (Beijing, China). The raw data were deposited in GenBank under the BioProject PRJNA808047 and BioSample SAMN26022686. The real-time quantitative PCR (RT-qPCR) was conducted using primers E2F1, SDS3, and Rad51 and reference gene primer *SW22934*. The reaction procedure included one cycle at 95 °C for 5 min, followed by 40 cycles at 95 °C for 10 s and at 60 °C for 30 s. Expression levels were calculated by the 2^−ΔΔt^ values method using three replicates.

## 3. Results

### 3.1. The Nbrbls Identified in N. bombycis Genome

A total of 52 *Nbrbl*s were identified in the *N. bombycis* genome, composing the largest protein family in *N. bombycis*. As shown in Table 2, the *pI* of different NbRBLs was variable, ranging from 4.55 to 9.13. The molecular weight of the NbRBLs is from 20 to 35 kDa. Thirty of 52 NbRBLs contain a Ricin B lectin domain (RBLD). The deficiency of RBLD was also found in other microsporidia [14].

### 3.2. The NbRBLs Are High Divergent

**Figure 1:**
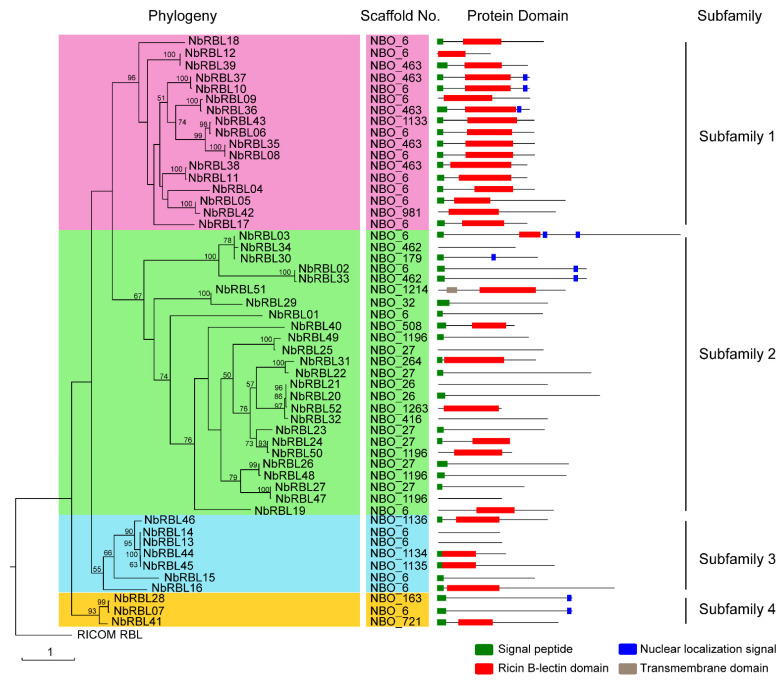
Phylogenetic analysis of NbRBLs. The phylogenetic tree was constructed using RaxML [24] with the Maximum Likelihood model from multiple sequence alignment of NbRBLs and visualized using the iTOL (http://itol.embl.de/, accessed on 15 September 2021). The NbRBL family was divided into four subfamilies. Branches in same background color indicate members in a subfamily. RICOM RBL, the castor ricin B chain (GenBank accession no, ACY38598.1), was used as the out group.

Phylogeny analysis shows a high level of divergence among the NbRBLs, which can be grouped into 4 subfamilies containing 17, 25, 7, and 3 members (Figure 1). Subfamily 1 is relatively conserved in that all members contain the RBLD, 14 of which encode a signal peptide (SP). Some members of the other subfamilies have lost RBLD, suggesting that these members are more differentiated. In subfamily 2, 17 out of 25 members have lost the RBLD, and only half retain the SP. Again, in subfamily 3, some members show a loss of the SP, RBLD. All members of subfamily 4 have the SP, while only one has the RBLD. Furthermore, there were 9 members with a SP and a nuclear localization signal (NLS), indicating that these factors could be secreted into host nucleus. In summary, the NbRBLs are a highly differentiated protein family that may have diverse functions in parasites.

### 3.3. Expansive Mechanisms of the NbRBL Family 

**Figure 2:**
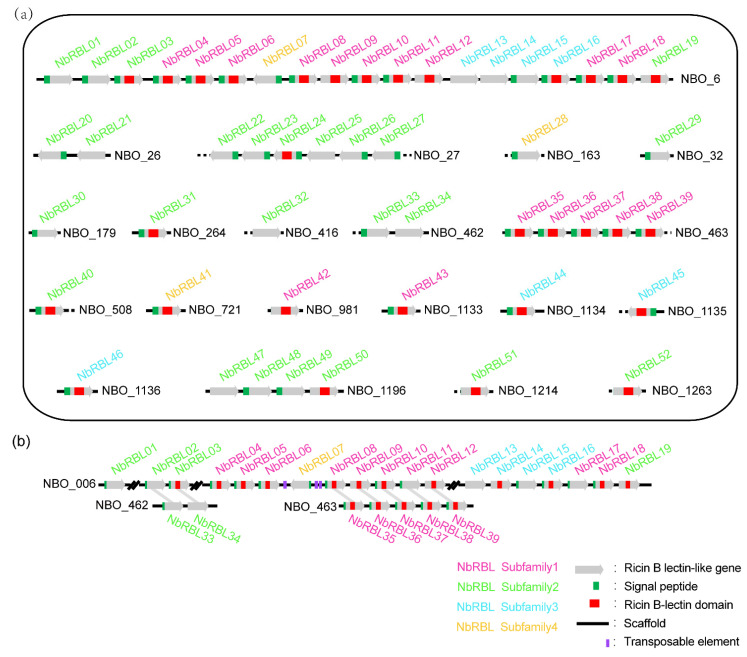
Chromosomal distribution of *Nbrbls*. (**a**) The 52 *Nbrbls* were localized to specific scaffolds based on the whole-genome sequences of the *N. bombycis* CQ1. A different color represents a different NbRBL subfamily. On the NBO_6 Scaffold, each NbRBL subfamily members were distributed. (**b**) Syntenic distributions of *Nbrbls* between scaffolds NB0_6, NB0_462, and NB0_463.

By mapping *N. bombycis* genome [17], we found that the 52 *Nbrbls* are located on 20 scaffolds (Figure 2a). Tandem gene duplications were found on scaffolds NBO_6, NBO_27, NBO_463, and NBO_1196, containing 19, 6, 5, and 4 genes, respectively. Moreover, we found that the members of each NbRBL subfamily were distributed on the different scaffolds. Members of each NbRBL subfamily were distributed on the NBO_6, but on the NBO_463, there only existed members of subfamily 1, and on the NBO_27 and NBO_1196 Scaffold, there were only members of subfamily 2. The NbRBL family formed clusters in the *N. bombycis* genome, indicating that the *Nbrbl*s experienced large-scale duplication. In the largest region containing the tandem duplications (TDs) and segmental duplications (SDs) of *Nbrbl*, we found transposable elements (TEs) flanking the SD region (Figure 2b). 

### 3.4. The Reduction of Key Motifs in NbRBL

**Figure 3:**
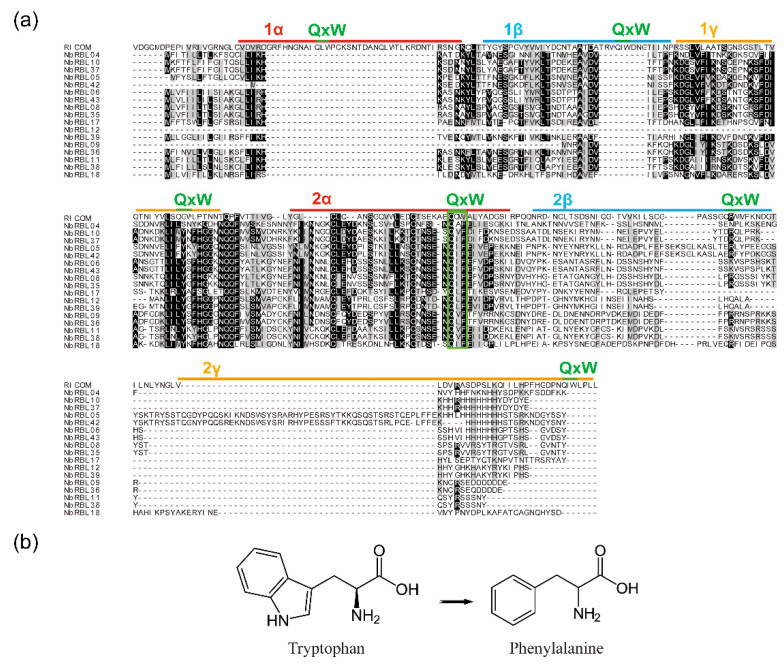
Multiple sequence alignment of NbRBL subfamily 1. (**a**) NbRBL subfamily 1 has three distinct subdomains: α, β, and γ, which have same structure as the RTB of castor. However, there is no obvious QxW motif in α subdomain, and the QxW motif in α subdomains turns into QxF motif (the green box). (**b**) Amino acid molecular structure of tryptophan and phenylalanine. Compared with tryptophan, the molecular weight of phenylalanine is smaller, and the structure becomes simpler.

The Ricin B lectin domain has been referred to as the (QxW)_3_ domain and the three homologous regions as the QxW repeats. Through multiple sequence alignment of NbRBL family, it is found that family 1 is relatively conservative in all subfamilies. Compared with RTB of castor, subfamily 1 also has three distinct subdomains: α, β, and γ. However, there is no obvious QxW motif in the α subdomain, even the QxW motif in α subdomains turns into QxF motif, which is found in three other three families (Figure 3 and Figures S1–S3).

**Figure 4:**
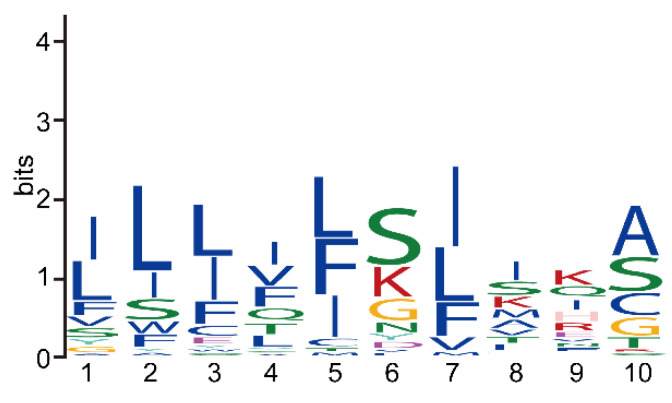
The SP motif of NbRBL family. Conserved motifs of NbRBL SPs analyzed using MEME tools. The conserved amino acid sequence [ILF][LI][LIF][IV][LFI][SK][IL]IK[ASC] in the SP of NbRBL protein, demonstrated that NbRBL proteins had similar secretion pathways.

We also analyzed the sequence feature of the SPs in NbRBL and found that their lengths ranged from 12 to 24 amino acids. A conserved amino acid motif [ILF][LI][LIF][IV][LFI][SK][IL]IK[ASC] was predicted, demonstrating that NbRBLs have similar secretion pathways (Figure 4). In addition, some researchers have found that there is a conserved amino acid sequence PEXEL/VTS/HT at the N-terminus of most secreted proteins of *Plasmodium*, which enable secreted proteins to pass through the vacuole [1,31]. We found a conserved amino acid sequence in the SP of NbRBL family members. It is speculated that they have similar secretory pathway and are secreted into host cells to play a regulatory role.

### 3.5. Expressions of Nbrbls during Infection

**Figure 5:**
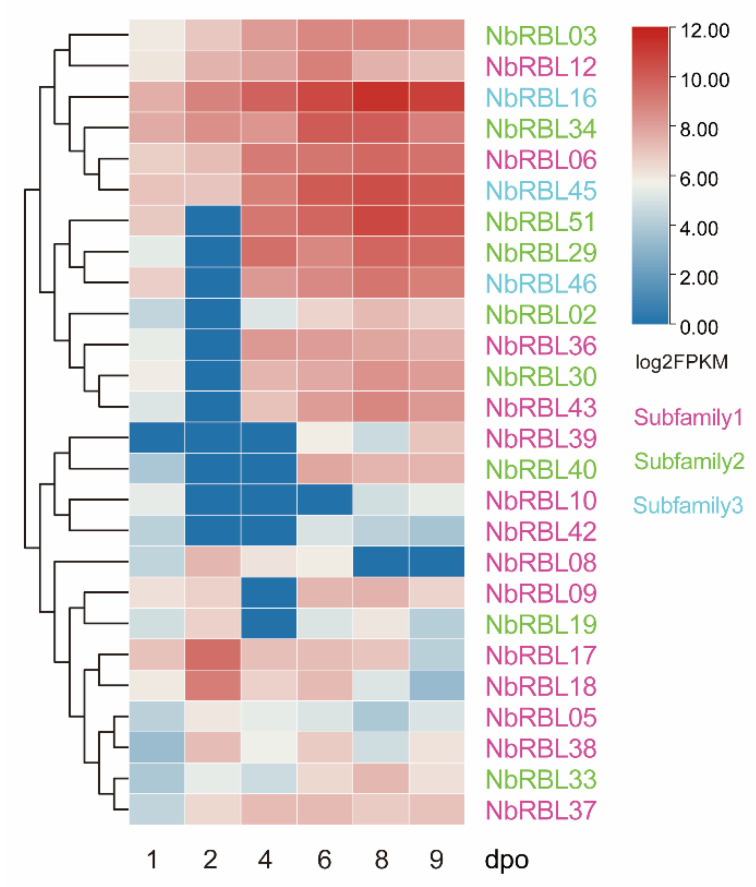
Expression patterns of *Nbrbls* in the *B. mori* embryo infected by *N. bombycis*. The expression level of *Nbrbls* in *N. bombycis*-infected embryo of *B. mori* after 1–9 days post oviposition (dpo) was calculated from the RNA-Seq data we published before [30]. Each column represents a time-point, each row represents a gene. For detailed FPKM of *Nbrbl*s, see Table S1.

**Figure 6:**
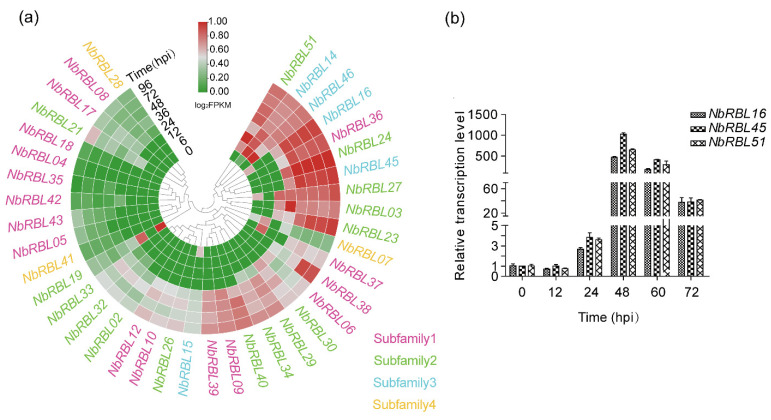
Expression of the *Nbrbls* in the *BmE-SWU1* cells infected with *N. bombycis*. (**a**) RNA-seq analysis of *Nbrbl*s expression in *N. bombycis*- infected the *BmE-SWU1* cells. Each column represents a time-point, each row represents a gene. The quantification of *Nbrbl* expressions was shown in Table S2. (**b**) RT-PCR examination of expression profiles of the *Nbrbls* in *BmE-SWU1* infected with *N. bombycis*.

*N. bombycis* can be transmitted vertically from infected females to eggs, resulting in congenital infections in embryos. Based on the RNA-seq data from articles published in the scientific literature [30], we analyzed the expression patterns of *Nbrbls* in *B. mori* embryos infected with *N. bombycis* and found that 26 of the identified *Nbrbls* were expressed during infection in *N. bombycis*. No expression of NbRBL subfamily 4 was detectable, Members of all other subfamilies showed expression during infection. Among them, 14 of 17 members in subfamily 1 were expressed. In addition, we found that three *NbRBLs* from subfamily 2 (*Nbrbl51*) and subfamily 3 (*Nbrbl16* and *Nbrbl45*) were highly expressed. Apart from *Nbrbl06,* most of the members of subfamily 1 showed a lower level of expression. Five genes from subfamily 1 (*Nbrbl05, Nbrbl08, Nbrbl09 Nbrbl17,* and *Nbrbl18*) were highly expressed early on and down-regulated during embryos development (Figure 5). We further analyzed the expression patterns of *Nbrbls* in the infected *BmE-SWU1* cells. The results showed that the overwhelming majority of *Nbrbls* were expressed and *Nbrbl16*, *Nbrbl45,* and *Nbrbl51* were highly expressed (Figure 6a). Then, we selected three genes (*Nbrbl16, Nbrbl45,* and *Nbrbl51*)*,* highly expressed to examine their expression profile in the *BmE-SWU1* cells after *N. bombycis* infection (Figure 6b). The data showed that the expression of these three genes were up-regulated at 48 hpi. 

### 3.6. Subcellular Localization of NbRBL16 and NbRBL28

**Figure 7:**
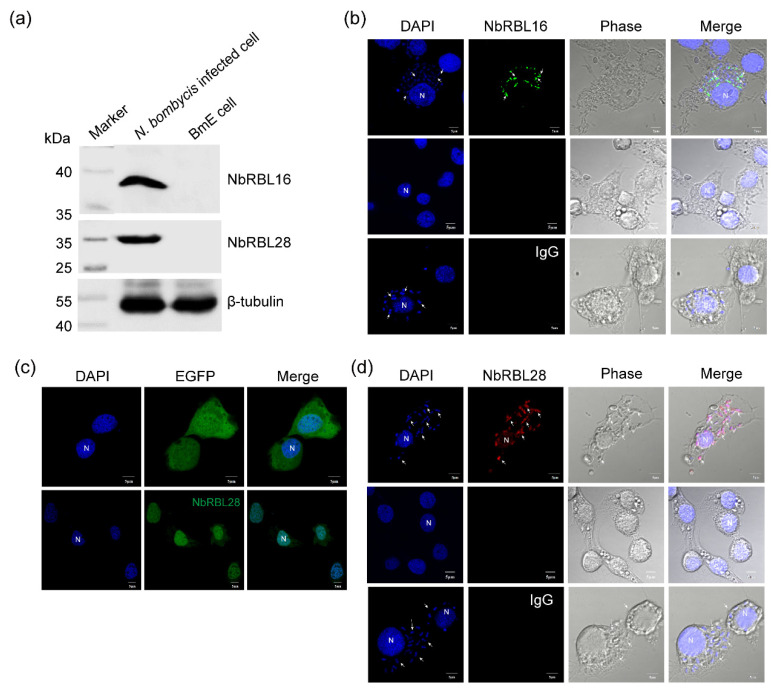
Immunoblot and IFA detecting the subcellular localization of NbRBL16 and NbRBL28 in an infected cell. (**a**) Specific detection of the NbRBL16 and NbRBL28 antibody. Proteins extracted from *N. bombycis*-infected cells were subjected to Western blot using polyclonal antibody against NbRBL16 and NbRBL28. (**b**) BmE-SWU1 cells were infected with *N. bombycis* at 48 h. Cells were fixed and stained with DAPI (DNA-specific dye, blue), anti-NbRBL16 (green) antibodies, and the mouse IgG as control. The uninfected BmE-SWU1 cells were incubated with anti-NbRBL16 (green) antibodies as the control. (**c**) The subcellular localization of NbRBL28::EGFP in BmE-SWU1 cell. (**d**) BmE-SWU1 cells were infected with *N. bombycis* at 48 h. Cells were fixed and stained with DAPI DNA-specific dye (blue), anti- NbRBL28 (red) antibodies and the mouse IgG as control. The uninfected BmE-SWU1 cells were incubated with anti-NbRBL28 (red) antibodies as control. The white arrowheads indicate *N. bombycis*. Bars, 5 μm.

First, we verified the specificity of the antibody using Western blotting, which revealed that the antibody of NbRBL16 and NbRBL28 distinguishes these endogenous proteins from the total proteins in *N. bombycis* infected cell (Figure 7a). The NbRBL16 protein is located in the cytoplasm of schizont in the proliferating stage while the mature spores gave no fluorescent signal (Figure 7b), similar to that of Cyto-NbHsp70 [28]. NbRBL28 contains an N-terminal signal peptide and nuclear localization signal (NLS) sequences (Figure 1), which was co-expressed with EGFP in *BmE-SWU1* to assess whether it could be secreted into the host nucleus. As expected, we found that four NbRBL28 proteins could be located in the *BmE-SWU1* cell nucleus (Figure 7c). Although NbRBL28-EGFP fusion protein was located in the host nucleus, NbRBL28 could be secreted into the host nucleus during *N. bombycis* infection. The result showed that NbRBL28 was not only located in the cytoplasm of the schizont, but was also detected in the host cell nucleus (Figure 7d), which demonstrated that NbRBL28 was a secreted protein targeted to the host cell nucleus.

### 3.7. NbRBL28 Regulates Gene Expressions Involved in Host Cell Cycle

**Figure 8:**
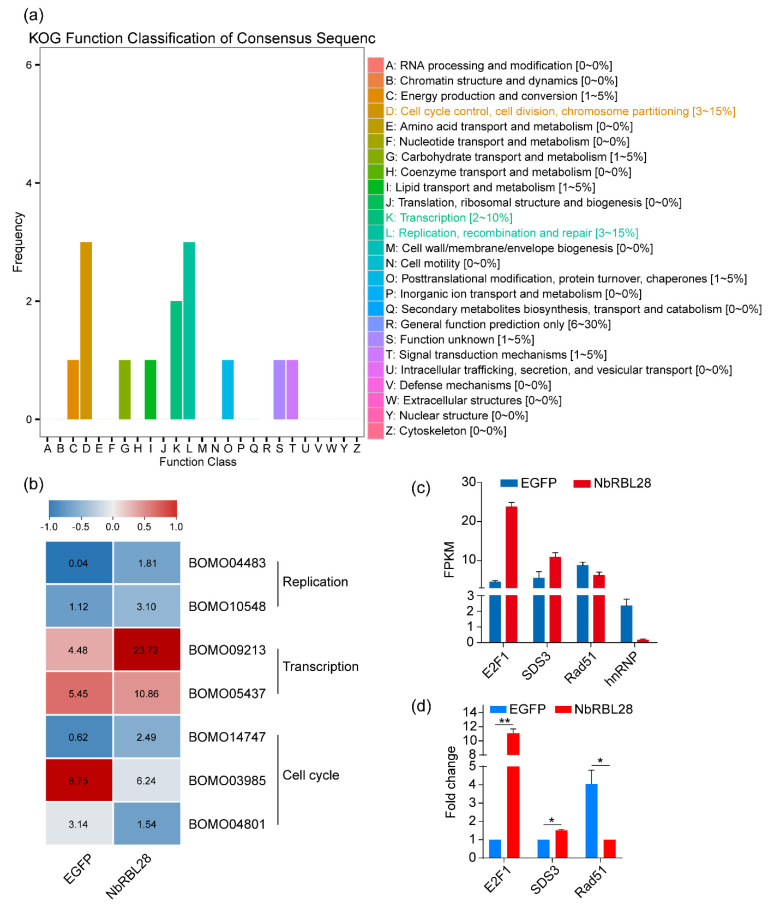
NbRBL28 alters the host cell transcriptome. (**a**) KOG function classification of the differentially expressed genes when comparing RBL28 versus EGFP-transfected cells. (**b**) Heatmap representation of the genes identified by KEGG analysis of the different data sets. (**c**,**d**) RT-qPCR analysis for differently expressed genes were coincidental with those of RNA-Seq. *, *p* < 0.05; **, *p* < 0.01.

Since NbRBL28 was detected in the infected host cell nucleus, it was likely to account for the gene expression changes triggered by infection. To verify this hypothesis, we performed a transcriptomic analysis of the BmE-SWU1 cells transfected with *Nbrbl28::egfp-* and *egfp-*containing plasmids. Filtered data were presented in Table S3. To identify which pathways were differently changed, we performed KOG and KEGG pathway analysis. KOG analysis revealed that a number of cell cycle, cell division processes, as well as replication transcription processes, were enriched (Figure 8a). KAGG pathway analysis revealed an enrichment of 11 pathways with *p* < 0.05 in BmE-SWU1 expressing *Nbrbl28::egfp*. Genes involved in transcriptional regulation (*E2F1* and *SDS3*) were up-regulated, while a gene (*Rad51*) functioning in DNA repair was down-regulated (Figure 8b,c). The E2F1 is a transcription factor involved in transformation of cell cycle from G1 phase to S phase [32,33]. The *Rad51* participates in the cell cycle, replication and repair [34,35]. In summary, these data indicated that NbRBL28 was positively regulating the expression of host cell genes involved in controlling the cell cycle progression.

## 4. Discussion

Ricin B-lectin domain proteins have been identified in bacteria, fungi, plants, invertebrates, and higher animals. Examples include Xylanase in *Streptomyces*, Ricin in the Castor bean, lactose-binding lectin in earthworms, the mannose-receptor in macrophages and RsA in *Rhizoctonia solani* [36]. RBL has been identified in most genera of microsporidia, including *Anncaliia*, *Encephalitozoon*, *Nematocida,* and *Spraguea* [14,37]. Because of its broad distribution and presence in the microsporidia, it is speculated that this gene family predates microsporidia evolution. Encephalitozoon, which has a highly reduced genome, still retains this gene family [38]. We have here identified 52 *Nbrbls,* which is the largest gene family in *N. bombycis*. Further study of RBL is helpful to understand the evolution of microsporidia gene and the relationship between microsporidia and its host.

Members of this family form clusters in the genome of microsporidia. In *E. cuniculi*, four *rbl*s are located on a single syntenic block, and six of the eight *rbls* of *N. ceranae* are found in NCER_1015 [14]. In *N. bombycis*, NBO_0006 was at the core of the gene family, and *rbl* was most likely the first to appear in this region. In addition, most of the genes appeared in pairs, and there were a large number of duplicate genes. The animal-derived “piggyBac” DNA transposons were found the in genome of *N. bombycis.* These mobile genetic elements encode functional transposases that are capable of recognizing a specific TTAA motif, which it cleaves to insert itself across different regions of the genome [39]. These results indicated that transposable elements most likely played an important role in mediating the duplication of *Nbrbls*. It is speculated that *N. bombycis* has a small number of *rbl*s on a single syntenic block as *E. cuniculi* in the early stage of evolution, and then gene amplification events such as tandem repeat and fragment repeat in the process of evolution occurred. Gene duplication is very important in the evolution of organisms, and gene duplication and differentiation have been considered as the driving force for a gene to produce new functions. These may suggest that the family would obtain new functions through gene replication and non-synonymous mutation to adapt to the changing living environment.

Our work also showed that evolutionary divergence has also occurred among microsporidia RBL protein genes. First of all, the number of *rbl* in different microsporidia varies tremendously. There are only 4 *rbl*s in *E. cuniculi* and 52 *rbl*s in *N. bombycis*. However, *rbl* has not been identified in *N. parisii* genome [11]. Secondly, some members of RBL protein family of microsporidia lost their RBLD. The 52 NbRBLs can be classified into 4 subfamilies with phylogenetic analysis. Subfamily 1 is relatively more conserved as all members have a RBLD and most proteins have SP, indicating that the NbRBLs of subfamily 1 retain an original galactose-binding function. There is big difference among subfamily 1 and other subfamilies, in which some members lost the RBLD. It showed that the sequences of NbRBL varied greatly, so that likely became a multi-functional family. Compared with Ricin B lectin, the motif of NbRBL turned into QxF from QxW, which was also found in the RBLs of *Anncaliia algerae* [16]. Phenylalanine was replaced by tryptophan, both of which were hydrophobic amino acids. Compared with tryptophan, the molecular weight of phenylalanine is smaller, and the structure becomes simpler. It is suggested that this was a kind of reduction that happened at the amino acid level in microsporidia. Besides, this motif substitution may alter the selectivity of RBLs for specific glycoproteins on host cytoplasm membrane, which are important for the parasite infection. 

Previous studies have shown that *Nbrbl* (identified as *Nbrbl03* in our study) was highly transcribed after 42 hpi [15], Our results showed that *Nbrbl03* was highly expressed after 12 hpi in BmE-SWU1 cells. We also found that *Nbrbl14*, *Nbrbl46,* and *Nbrbl51* were also highly expressed at 6 hpi, but some *Nbrbls* (such as *Nbrbl04*, *Nbrbl05*, *Nbrbl17,* and *Nbrbl18*, etc.) were expressed at low levels in the infected BmE-SWU1 cells. Interestingly, *Nbrbl17* and *Nbrbl18* were high level expressed in the infected embryos, which suggested that different NbRBLs may have different biological functions. 

Interestingly, NbRBL28 was the first RBL member that was found to be secreted into the host nucleus and likely to modulate the host cell cycle. This modulation model was also reported in other intracellular pathogens [40,41], for instance, *T. gondii* secrete GRA16, GRA24, ROP16, and TgIST into the host nucleus to interfere with gene expressions [42,43,44,45,46,47,48]. Besides, there are 13 NbRBLs without a SP, of which 9 were predicted to be located in the nucleus (Table 2), suggesting that these members may regulate the gene expressions of the parasites themselves. Furthermore, it has been reported that lectins have diverse roles in parasites, and can mediate adhesion of the parasite to the host cell [49]. For example, the NbRBL03 was reported to enhance spore adhesion to the host cells [15], and that NbRBL51 is an only member, containing a transmembrane domain, indicating that it is a membrane protein and most likely promotes adhesion too. Similar to the RTB, secreted and transmembrane NbRBLs likely bind to glycoproteins on the host cytoplasm membrane to mediate the adhesion. Therefore, NbRBLs play important and multiple roles during infection and pathogen development. 

In summary, we primarily discussed identification, phylogenetic classification, molecular evolution, and gene expression analyses of the NbRBL gene family. The increase of NbRBL genes suggested that certain members have evolved to carry out a larger number of functions to adapt to intracellular life. Therefore, RBL, which is an ideal target, holds significance to the study of microsporidium gene evolution and the analysis of the mechanism of interaction between microsporidium and host.

## Figures and Tables

**Table 1:** PCR primers.

Gene Name	Forward Primers (5′ to 3′)	Reverse Primers (5′ to 3′)
*Nbrbl16*	GTTCTGTCAATCCAAGTGTTCC	ACTGTGCTTAGAAAGACGATCA
*Nbrbl45*	TCCTGTTGATCAAAACGTTGTC	TGAGTGTGGTGTATATCGTCAG
*Nbrbl51*	TGTGTCTACGTGTGTCGATAAA	TCAAGAGAACCAGCAGTAAGAC
*Nbtubulin*	CTGGGGATAGTATGATCGCAAGA	CACAGCATCCATTGGAAACG
*E2FI*	GAAATCTTCACAGAACGGAGTG	AGAACGTTCGTGATGTCGTATA
*SDS3*	AAACATCTCAACTCTGGCAGTA	CCTTATTCGTTCCACATTCGTC
*Rad51*	ACAGTCGTCCCACAGCAA	CGATGAGGCAGTGTAGGT
*SW22934*	TTCGTACTGGCTCTTCTCGT	CAAAGTTGATAGCAATTCCCT

**Table 2:** The NbRBLs identified in *N. bombycis* genome.

NbRBLs	Locus ID	GenBank Accession No.	Amino Acid Residues (Aa)	Molecular Weight (Da)	pI	Signal Peptide	Subcellular Localization	Domain
NbRBL01	NBO_6:56729..57397:+	ON211418	222	25,337.44	7.56	1-13	cytosol	NA
NbRBL02	NBO_6g0014	ON211419	315	36,999.70	7.96	1-17	nucleus	NA
NbRBL03	NBO_6g0015	ON211420	514	57,702.24	6.00	1-15	nucleus	Ricin B-lectin
NbRBL04	NBO_6g0041	ON211421	205	23,554.40	9.09	1-14	nucleus	Ricin B-lectin
NbRBL05	NBO_6g0043	ON211422	270	31,416.47	7.74	1-16	nucleus	Ricin B-lectin
NbRBL06	NBO_6g0045	ON211423	204	22,808.19	7.26	1-14	mitochondria	Ricin B-lectin
NbRBL07	NBO_6g0046	ON211424	285	32,927.32	9.00	1-20	nucleus	NA
NbRBL08	NBO_6g0047	ON211425	205	22,803.26	8.99	1-14	extracellular	Ricin B-lectin
NbRBL09	NBO_6g0048	ON211426	153	17,934.29	4.70	No	nucleus	Ricin B-lectin
NbRBL10	NBO_6g0049	ON211427	194	22,864.24	6.25	1-14	nucleus	Ricin B-lectin
NbRBL11	NBO_6:123114..123683:−	ON211428	189	21,566.63	8.76	1-16	extracellular	Ricin B-lectin
NbRBL12	NBO_6g0050	ON211429	115	13,064.90	8.63	No	cytoskeleton	Ricin B-lectin
NbRBL13	NBO_6:131790..132149:−	ON211430	119	13,888.14	6.32	No	nucleus	NA
NbRBL14	NBO_6g0058	ON211431	123	14,260.48	6.39	No	nucleus	NA
NbRBL15	NBO_6g0060	ON211432	205	23,463.03	8.99	1-15	nucleus	NA
NbRBL16	NBO_6gi003	ON211433	374	42,355.07	6.35	1-15	nucleus	Ricin B-lectin
NbRBL17	NBO_6g0061	ON211434	189	22,357.92	5.74	1-17	nucleus	Ricin B-lectin
NbRBL18	NBO_6g0062	ON211435	224	25,993.40	6.83	1-14	Golgi complex	Ricin B-lectin
NbRBL19	NBO_6g0108	ON211436	245	26,625.59	8.65	No	nucleus	Ricin B-lectin
NbRBL20	NBO_26:48175..49206:+	ON211437	343	37,408.14	8.46	1-18	extracellular	NA
NbRBL21	NBO_26g0023	ON211438	230	24,843.75	6.49	No	nucleus	NA
NbRBL22	NBO_27:44489..45466:+	ON211439	325	35,631.08	5.67	1-14	extracellular	NA
NbRBL23	NBO_27g0016	ON211440	342	37,076.99	6.18	1-15	extracellular	NA
NbRBL24	NBO_27g0018	ON211441	259	28,920.21	8.68	1-12	extracellular	Ricin B-lectin
NbRBL25	NBO_27:50577..51263:+	ON211442	228	26,353.80	5.90	No	mitochondria	NA
NbRBL26	NBO_27g0019	ON211443	278	30,457.43	9.04	1-23	extracellular	NA
NbRBL27	NBO_27g0020	ON211444	322	35,330.53	8.64	1-12	extracellular	NA
NbRBL28	NBO_163g0001	ON211445	285	32,984.50	9.13	1-20	nucleus	NA
NbRBL29	NBO_32g0011	ON211446	233	26,695.26	8.92	1-27	nucleus	NA
NbRBL30	NBO_179g0001	ON211447	211	24,218.89	5.12	1-15	nucleus	NA
NbRBL31	NBO_264:2462..3085:−	ON211448	207	23,435.10	8.81	1-12	nucleus	Ricin B-lectin
NbRBL32	NBO_416g0002	ON211449	232	25,229.27	7.74	No	nucleus	NA
NbRBL33	NBO_462g0008	ON211450	315	36,938.62	7.96	1-17	nucleus	NA
NbRBL34	NBO_462g0009	ON211451	173	18,373.24	7.84	No	nucleus	NA
NbRBL35	NBO_463g0001	ON211452	205	22,747.16	8.99	1-14	extracellular	Ricin B-lectin
NbRBL36	NBO_463g0002	ON211453	194	22,561.94	5.71	1-22	endoplasmic reticulum	Ricin B-lectin
NbRBL37	NBO_463g0004	ON211454	194	22,912.32	6.30	1-14	nucleus	Ricin B-lectin
NbRBL38	NBO_463g0005	ON211455	189	21,464.43	8.76	1-14	extracellular	Ricin B-lectin
NbRBL39	NBO_463g0006	ON211456	190	21,675.99	9.06	1-23	extracellular	Ricin B-lectin
NbRBL40	NBO_508g0001	ON211457	162	18,502.31	9.07	1-20	extracellular	Ricin B-lectin
NbRBL41	NBO_721g0001	ON211458	255	29,309.34	9.10	1-20	nucleus	Ricin B-lectin
NbRBL42	NBO_981g0001	ON211459	249	28,931.56	8.28	No	nucleus	Ricin B-lectin
NbRBL43	NBO_1133g0001	ON211460	204	22,731.15	7.23	1-14	extracellular	Ricin B-lectin
NbRBL44	NBO_1134:2994..3426:+	ON211461	144	16,418.60	6.81	1-24	extracellular	Ricin B-lectin
NbRBL45	NBO_1135g0001	ON211462	247	28,315.53	6.74	1-24	extracellular	Ricin B-lectin
NbRBL46	NBO_1136g0001	ON211463	232	26,654.78	8.78	1-12	mitochondria	Ricin B-lectin
NbRBL47	NBO_1196:1..366:−	ON211464	122	12,946.28	4.55	No	nucleus	NA
NbRBL48	NBO_1196:1677..2495:−	ON211465	272	29,582.35	8.93	1-17	mitochondria	NA
NbRBL49	NBO_1196:2983..3561:−	ON211466	192	22,211.52	7.63	1-15	mitochondria	NA
NbRBL50	NBO_1196:4918..5391:−	ON211467	157	17,024.65	5.00	No	cytosol	Ricin B-lectin
NbRBL51	NBO_1214g0002	ON211468	270	31,043.06	8.89	No	endoplasmic reticulum	Ricin B-lectin Transmembrane
NbRBL52	NBO_1263:1090..1494:+	ON211469	135	14,919.37	5.24	No	cytosol	Ricin B-lectin

## Data Availability

The raw data was deposited in GenBank under the BioProject ID PRJNA808047 and BioSample accession SAMN26022686.

## Supplementary Materials

The following supporting information can be downloaded at: https://www.mdpi.com/article/10.3390/jof8060551/s1, Figure S1: Multiple sequence alignment of NbRBL subfamily 2; Figure S2: Multiple sequence alignment of NbRBL subfamily 3; Figure S3: Multiple sequence alignment of NbRBL subfamily 4; Table S1: Data of NbRBLs in the *B. mori* embryo infected by *N. bombycis*; Table S2: Data of NbRBLs in the BmE-SWU1 infected by *N. bombycis*; Table S3: RNA-Seq of NbRBL28 expressed in BmE-SWU1.
