# Supplementary material for: Characterization of the Largest Secretory Protein Family, Ricin B Lectin-like Protein, in Nosema bombycis: Insights into Microsporidian Adaptation to Host
Source: J Fungi (Basel). 2022 May 24;8(6):551. doi: 10.3390/jof8060551 (PMC9224602; doi:10.3390/jof8060551)
Supplement: Supplementary file 1 [file jof-08-00551-s001.zip › jof-1732713-supplementary-Figures S1¿CS3.pdf]

Figures S1–S3

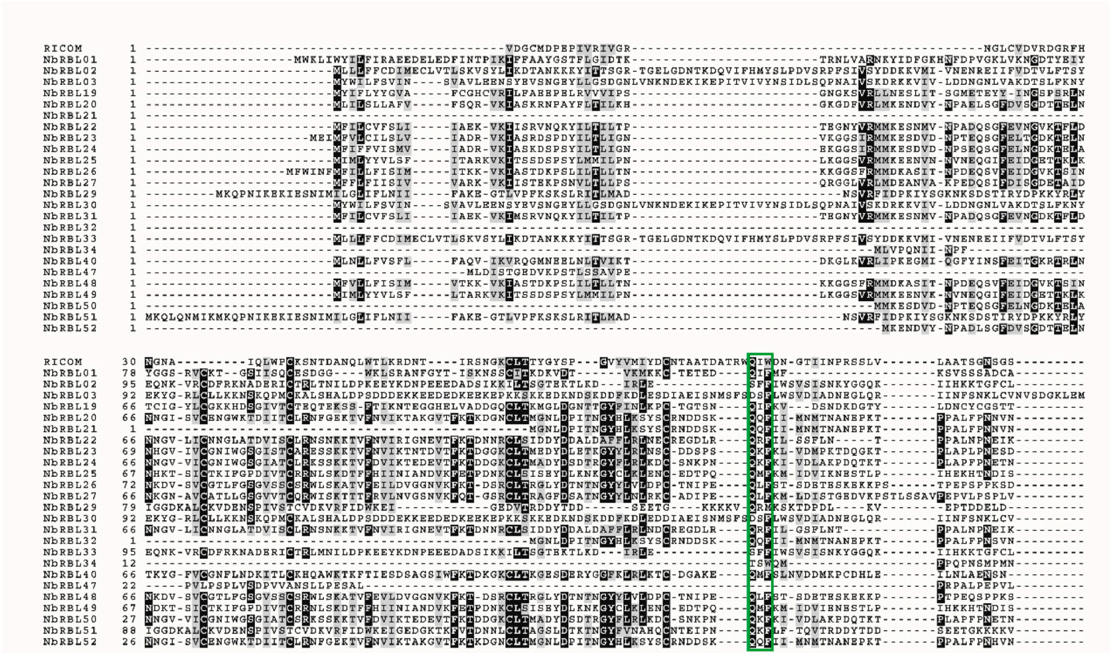

Figure S1. Multiple sequence alignment of NbRBL subfamily 2. In the NbRBL subfamily 2, there is no obvious QxW motif in α subdomain, and the QxW motif in α subdomains turns into QxF motif (the green box).

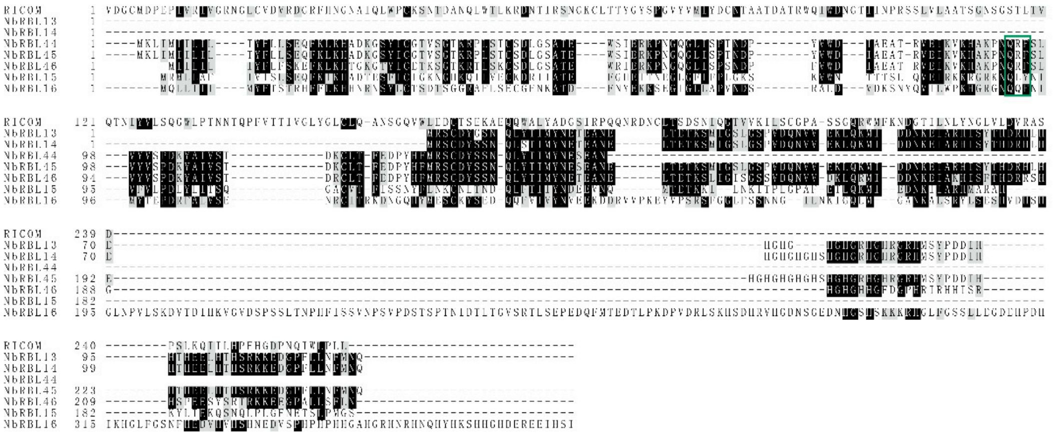

Figure S2. Multiple sequence alignment of NbRBL subfamily 3. In the NbRBL subfamily 3, there is no obvious QxW motif in α subdomain, and the QxW motif in α subdomains turns into QxF motif (the green box).

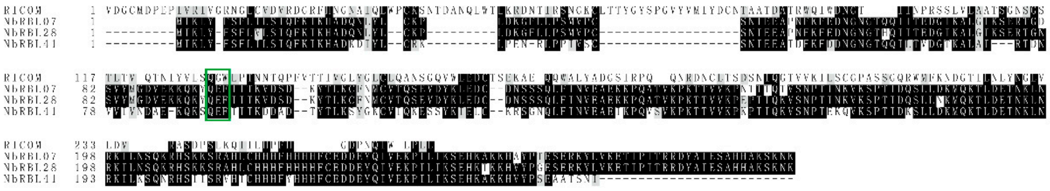

Figure S3. Multiple sequence alignment of NbRBL subfamily 4. In the NbRBL subfamily 4, there is no obvious QxW motif in α subdomain, and the QxW motif in α subdomains turns into QxF motif (the green box).
